# The Importance of a Relationship-Centred Approach to Deprescribing for People with Dementia or Mild Cognitive Impairment in Primary Care: A Qualitative Study

**DOI:** 10.1177/14713012251376227

**Published:** 2025-11-20

**Authors:** Nicola Andrews, Cindy Brooks, Jay Amin, Rosemary Lim, Michele Board, Sue Latter, Simon Fraser, Kinda Ibrahim

**Affiliations:** 1 School of Health Sciences, University of Southampton, UK; 2 National Institute for Health and Care Research (NIHR) Applied Research Collaboration Wessex, Southampton, UK; 3 Memory Assessment and Research Centre, 7426 Hampshire and Isle of Wight Healthcare NHS Foundation Trust, Southampton, UK; 4 School of Clinical and Experimental Sciences, Faculty of Medicine, University of Southampton, UK; 5 Reading School of Pharmacy, University of Reading, UK; 6 Ageing and Dementia Research Centre, Bournemouth University, Dorset, UK; 7 School of Primary Care, Population Sciences and Medical Education, Faculty of Medicine, University of Southampton, UK; 8 Primary Care Research Centre, University of Southampton, UK

**Keywords:** dementia, mild cognitive impairment (MCI), deprescribing, primary care, qualitative research

## Abstract

Polypharmacy (taking five or more regular medications) is common in people with dementia or mild cognitive impairment (MCI) and is associated with poor outcomes such as decline in cognitive and physical functioning, falls and hospital admission. Reducing or stopping unnecessary medications (deprescribing) can help improve outcomes but limited research has been undertaken with people with dementia or MCI, especially in primary care. This study explored the perspectives and experiences of people with dementia or MCI, informal carers and healthcare professionals on deprescribing decision-making in this setting. Seven people with dementia, two people with MCI and nine informal carers took part in photo elicitation and an in-person interview. They took photographs of their day-to-day management of medication, which were used during the interview to probe the conversation. Sixteen healthcare professionals, including general practitioners, pharmacists, nurses, social prescribers and National Health Service memory clinic professionals, participated in individual online interviews. Data was analysed using inductive thematic analysis. Findings suggest the need to navigate ‘patient vs carer vs professional voices’ in the deprescribing process to ensure everyone’s voice is included. It should take into account patient cognitive abilities, autonomy and independence, as well as carer experiences and the need for a written summary of what has been discussed and agreed. Flexible, tailored, cross-system medication management processes are needed to support effective deprescribing, including proactive follow-up. To facilitate deprescribing discussions, factors such as professional knowledge, safe environment, and sufficient time should be considered. Finally, careful consideration should be given to the potential impact of deprescribing decisions on day-to-day medication management and carer burden. These findings provide novel insights that demonstrate the need for a relationship-centred approach for deprescribing for people with dementia or MCI, which should inform future research on development of a primary care deprescribing intervention for this group of people.

## Introduction

People with dementia or mild cognitive impairment (MCI) frequently live with other long-term conditions and experience polypharmacy (Clague et al., 2017). Polypharmacy, commonly defined as taking five or more regular medications (Masnoon et al., 2017), is associated with increased risk of inappropriate medication, drug interactions and adverse effects including falls, cognitive decline, emergency department attendance, hospitalisation and death (Mueller et al., 2018; Onder et al., 2013). Research suggests that optimising medications through deprescribing (reducing or stopping medication) is safe, feasible and beneficial for older people (Iyer et al., 2008; Page et al., 2016; Thio et al., 2018; van der Cammen et al., 2014).

Many older adults with dementia are uncomfortable taking multiple medications, with a majority willing to have medication deprescribed (Growdon et al., 2022), highlighting an opportunity to reduce prescribing and improve quality of life for this vulnerable population. In the United Kingdom (UK), general practitioners (GPs), with support from pharmacists working in GP practices, are ideally positioned to facilitate deprescribing as they have access to an individual’s full medical history (including current medication, drug history, diagnoses and investigations) to help inform decisions about medications (Duncan et al., 2017). However, most deprescribing interventions for people with dementia or MCI have been limited primarily to long-term care or inpatient settings (Sawan et al., 2021). Yet, in the UK, around 61% of people with dementia live at home with medication being part of day-to-day living (Prince et al., 2014).

Decisions relating to deprescribing are a responsibility shared by the older person and healthcare providers (Ontario Deprescribing in Long Term Care Stakeholder Team, 2020). Such shared decision-making is a collaborative process involving the individual and their healthcare professional working together to reach a joint decision about care (National Institute for Health and Care Excellence, 2021). It is central to ensuring medication is only used when it is beneficial and aligns with a person’s preferences (Chenoweth, 2024). However, optimising medications among older people with dementia or MCI is complicated by reduced mental capacity, challenges in communication and difficulties establishing goals of care (Reeve et al., 2015), which necessitates involvement of informal carers (family or friends providing unpaid care). Limited evidence suggests they can both assist and complicate the process (Pel-Little et al., 2021). However, a narrative review of deprescribing effectiveness in people with dementia found limited evidence of involvement of either the person themselves or their carer in deprescribing interventions (Sawan et al., 2021). It is, therefore, essential to involve people with dementia or MCI and their informal carers in designing services and interventions that are focused on their needs by addressing these barriers.

Understanding the perspectives of people with dementia or MCI, their informal carers and relevant healthcare professionals, on preferred approaches to deprescribing is a key first step in developing recommendations for strategies to inform this practice in primary care. This study aimed to explore the views and experience of people with dementia or MCI, informal carers and healthcare professionals on deprescribing and the challenges and facilitators of deprescribing decision-making in primary care.

## Methods

### Study Design

An inductive qualitative interview design with photo elicitation was used to capture an in-depth understanding of participants’ perspectives.

### Recruitment

A purposive sample of people with dementia or MCI living in their own home, informal carers and healthcare professionals working in primary care or National Health Service (NHS) memory clinics (outpatient settings) was recruited. Maximum variation was sought within the sample in relation to characteristics including gender and age. Table 1 details the study’s inclusion and exclusion criteria.

**Table 1.** Participant Inclusion and Exclusion Criteria

	Inclusion criteria	Exclusion criteria
People with dementia or MCI	• Aged 55 or over. • Confirmed medical diagnosis of dementia or MCI. • Prescribed five or more regular medications. • Had mental capacity to understand the nature of the research and give consent to participate.	• Living in residential care (nursing home or residential home) or in accommodation with onsite care provision (e.g., extra-care housing). • In receipt of end-of-life care. • Did not wish to take photographs, with or without support.
Family carers	• Provided informal care regularly to someone aged 55 and over, with any stage of dementia or MCI, taking five or more regular medications and living in their own home.	• Did not live within the study catchment area.
Primary care healthcare professionals	• Provided care to people with dementia or MCI who take multiple medications in a primary care setting.	• Did not work in the study catchment area.
Healthcare professionals from NHS memory clinics	• Provided care to people who take multiple medications in an NHS memory clinic.	• Did not work in the study catchment area.

Participants with dementia or MCI were identified and invited to participate by two Participant Identification Centres (PICs), one an NHS memory research centre with a database of patients and informal carers who had expressed interest in research and the other an NHS memory clinic identifying patients receiving routine care from their caseload. Potential participants were contacted by telephone by a research nurse, with written information about the study sent by post to those that expressed interest. Informal carers were identified from the research centre database, via a participating patient, or from their response to an expression of interest poster shared with local dementia support groups. Participants were not intentionally recruited as patient-carer dyads. The study was advertised to healthcare professionals working in primary care or NHS memory clinics using a poster, shared via local professional networks and social media, inviting them to contact the research team to express interest in taking part. Professional recruitment was completely separate to recruitment of participants with dementia or MCI and informal carers.

### Data Collection

Each participant completed a single, semi-structured interview. Interviews were undertaken by experienced qualitative researchers (NA, CB) and explored the perceptions of participants and how deprescribing is best implemented for people with dementia or MCI. Interview topic guides are provided in Supplementary Materials.

For patients and informal carers, the interview was combined with photo elicitation and conducted in-person at the participant’s home. Photo elicitation is an interview technique where photographs are used to explore insights from participants and considered a useful approach for people with cognitive impairment (Lim et al., 2022). Participants were loaned a digital camera and asked to take photographs of anything that was related to managing their medications. Cameras were collected when participants had taken their photographs, the timeframe agreed with participants, mostly around one week later. The photographs were printed and used in the interview as cues for discussion. Interviews took place within two weeks following the camera collection. Interviews were audio-recorded using a digital voice recorder, transcribed verbatim and anonymised. Participants with dementia or MCI were offered the option to have a family member present for the interview to ensure their comfort.

Interviews with primary care and memory clinic professionals were conducted virtually using Microsoft Teams. Interviews were recorded via Microsoft Teams and the transcription facility used, with transcripts reviewed for accuracy shortly after the interview and anonymised.

### Data Analysis

Transcripts were analysed using inductive thematic analysis (Braun & Clark, 2022). Initial coding was completed independently by the two researchers who completed the respective interviews. NVIVO (version 14), Microsoft Excel and Microsoft Word were used to manage data during analysis. Patterns in the coded data across the two data sets were explored and insights from the interviews discussed with the Chief Investigator (KI), who is an experienced qualitative researcher, during two analysis meetings held whilst data collection was ongoing. Relationships between codes were mapped to generate an initial thematic map. These tentative themes were sense-checked and further refined with the study’s public and patient involvement and engagement (PPIE) group, consisting of two people with dementia and four informal carers.

The photographs were analysed on completion of data collection, grouped together under photographic themes. This analysis was both informed by and used to refine the thematic analysis. The final coded data corpus and the photographic analysis were reviewed at further meetings, with themes evolved through discussion and writing of theme narratives. The final thematic map was sense checked with the PPIE group, with their input and feedback yielding no new insights and confirming thematic saturation had been achieved.

All researchers who completed the analysis (NA, CB, KI) were female, had different professional backgrounds (a nurse, a medical sociologist and a pharmacist) and had considerable clinical and/or research experience with older people in primary care or community settings, including those living with dementia.

### Ethical Considerations

The study was approved by the London-Central Research Ethics Committee (23/PR/0366). All potential participants were provided with written study information and informed that participation was voluntary. All participants had capacity to consent and provided written, informed consent. People with dementia or MCI were invited to participate if experienced research nurses at the PICs assessed during initial telephone contact that they would have capacity to consent to the research. Capacity was assessed at the consenting visit and again prior to the interview by the researcher, who has experience of assessing mental capacity both in research and clinical roles. Mental capacity was assessed in line with the Mental Capacity Act 2005 Code of Practice (Department of Constitutional Affairs, 2007).

## Findings

Seven patients with dementia, two patients with MCI, nine informal carers and 16 healthcare professionals working in primary care (n = 13) or memory clinics (n = 3) participated in the study. Table 2 presents a summary of participant characteristics. Participants included four patient-carer dyads and at least two carers of individuals with dementia who did not have capacity to consent. Participants took 112 photographs in total, with a range of one to 21 per participant, carers taking photographs on behalf of three patients. Interviews ranged between 20 and 60 minutes in duration, with the average length of patient interviews being 30 minutes and the average length of both carer and professional interviews being 45 minutes. All but two patient interviews took place with a family member present, who sometimes prompted or clarified information provided by participants. Both of the patients interviewed without a family member present lived alone, with either no family or family who lived at a distance, one living with dementia and one with MCI.

**Table 2.** Participant Characteristics of Sample

Patients (n = 9)	
Gender	6 male, 3 female
Age	Range 66 to 87 years; median 80 years
Ethnicity	8 White British, 1 White other
Index of multiple deprivation decile^a^	Range 3 to 10; median 9 (10 = least deprived)
Number of regular medications^b^	Range 6 to 11; median 8
Lives alone	5 no, 4 yes
Carers (n = 9)	
Gender	8 female, 1 male
Age	Range 60 to 82 years; median 66 years
Ethnicity	All White British
Index of multiple deprivation decile^a^	Range 5 to 10; median 9 (10 = least deprived)
Relationship to person cared for	7 spouse, 1 child, 1 child’s partner
Lived with cared for person	7 yes, 2 no
Highest educational attainment	Degree level or above^c^ 3, professional qualification 4, GCSE or equivalent^d^ 1, No qualification 1
Professionals (n = 16)	
Professional role	7 GPs, 3 primary care pharmacist, 2 primary care nurse, 2 memory clinic doctor, 1 memory clinic nurse, 1 Social Prescriber^e^
Gender	13 female, 3 male
Age	5 aged 30 – 39, 8 aged 40 – 49, 3 aged over 50
Time since professional qualification	Range 5 to 38 years; median 12½ years
Length of time working in primary care / NHS memory clinic	Range 8 months to 33 years; median 5½ years
Primary care practice population size	Range 8000 to 60000^f^
Primary care practice deprivation level^a^	Decile range 4 to 10; median 7 (10 = least deprived)

^a^The Index of Multiple Deprivation is a UK measure of deprivation at small local area level based on seven domains including income and health.

^b^Regular medications included prescribed medication only.

^c^Degree level qualifications are further or higher education qualifications typically taken by students aged 18 and above.

^d^GCSE (General Certificate of Secondary Education) is a school-level academic qualification typically taken by students aged 14 to 16.

^e^A social prescriber is a healthcare professional working in primary care to connect people with non-medical support services to address social, emotional and practical needs.

^f^Primary care practices in the UK vary in size with differing numbers of GPs and patient populations. The average number of patients cared for by each fully qualified, full-time equivalent GP was 2258 in May 2025 (Royal College of General Practitioners, 2025).

**Figure 1.**
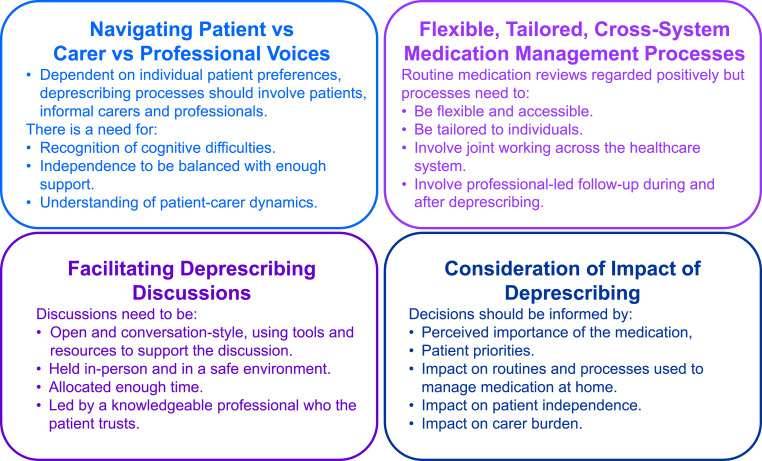
Overview of the Themes

Four main themes were generated to represent the data (Figure 1) and presented below. Photographic data primarily related to one theme and are presented alongside the quotes as supporting data for this theme.

### Navigating Patient, Carer and Professional Voices

Many professionals reported that patients with dementia or MCI were often taking multiple medicines and recognised the value of deprescribing.

It's really important that medications are rationalised and deprescribed where appropriate. (Professional 9)

However, some professionals raised concern about the potential for deprescribing to be perceived as withdrawal of care by a patient’s family, although many patients and carers were unconcerned about stopping medication.

Where it might become more tricky is when you're dealing with patient’s next of kin and/or an informal carer where they can see it as that you're withdrawing care. (Professional 6)

If somebody came along and said to me: ‘you don’t need that anymore’ then I would stop taking it and it wouldn’t worry me. (Patient 3)

Patients and carers frequently talked about trusting doctors and although willing to be led by professionals, patients and carers believed that they should be involved in decision-making about medication. Some patients and carers reported being included in decision-making discussions, others thought professionals made the decision and felt disempowered through a lack of information about their medicines.

They don’t say: ‘well, if you take this, this is what it does, if you don’t take it, then these are the consequences of you not taking it’, but nobody gives you that explanation. (Patient 1)

All groups of participants acknowledged that involving patients in discussions and decision-making requires recognition of the impact of their cognitive impairment and assessment of mental capacity. However, patients and carers reported how the impact of memory loss was frequently unrecognised in healthcare encounters. Memory problems associated with dementia and MCI mean that although the individual may have capacity to make decisions, these are made in the moment and information and decisions made can be lost, not remembered after the discussion.

If somebody was to say to him: “stop this now”, I’d need to know because that wouldn’t get taken out of the pot, unless whoever it was saying: “stop it” was in the room and saying: “right, we need to take these out”. It, it would just carry on and then by the time he came to fill the pots at the end of the week, he’d have forgotten, and it would all just go back in again. (Carer 1)

Yet, a lack of routine provision of written confirmation of what has been discussed and agreed during consultations, and carers’ difficulties accessing information instead of or as well as the patient, were identified as challenges when the patient attends appointments alone. Providing written information to take away after deprescribing discussions was perceived as particularly helpful for both patients and carers.

A letter advising me what is happening. What, what the likely outcome of the change is likely to be. (Patient 1)

Professionals acknowledged the importance of patient involvement. However, the extent to which this could be supported was shaped by considerations such as a patient’s preference to be involved in decisions and the patient’s overall health and wellbeing. Some professionals believed that it may not be appropriate to involve a patient if this would add additional stress or burden.

If [...] they are really upset if you talk about medication or you're going to make them distressed, then I think you should not involve the patient. (Professional 1)

Decision-making about deprescribing also needs to involve a carer or other advocate, who may have an essential role in managing medication at home.

I organise the delivery of the medication, I organise that carers give it to her so, if they have any questions, they come to me first. So, things like, like either should they use certain things or should they, you know, if they run out of something, so I do all of that. (Carer 5)

The valuable contribution carers can make to decision-making about medications might support both the patient and professionals. On one hand, professionals may not receive full or correct information without carer input, with carers often relied on to provide insights into a patient’s character, preferences and medical history. On the other hand, carers are able to relay information to patients following consultations to inform decisions to be made at a later date and ensure any changes are implemented.

It's helpful to have someone there who can give insight [... The patient] may not necessarily have knowledge or remember some of the key things that have happened to them, like a fall. (Professional 5)

However, involvement of carers is dependent on the extent to which the patient or the patient’s condition permits carer involvement.

Sometimes the patient themselves can be a barrier if they don't want them to be involved. (Professional 6)

Carers wanted to be involved, although many felt their voice was not always heard. For example, one, caring for her husband who lacked capacity to make decisions about medication, expressed frustration that she had limited influence as a Lasting Power of Attorney was not in place. Another spoke of numerous unsuccessful attempts to reduce medication by initiating discussions with professionals.

I’ve gone, you know: “can’t we get rid of some of these?”, you know. She’s on a stomach tablet because she takes so many tablets. [...] but they don’t. (Carer 9)

There were challenges to ensuring all voices were heard, however. Carers talked about the challenges faced in balancing their involvement with allowing patient independence and choices.

So, she’s very set on whether she’s taking [laxative] and whether she needs more or less [...], she thinks she knows but now she’s getting muddled up as to which day it was that she actually went to the loo [...] so, even that is getting a little bit more complicated. (Carer 8)

Professionals also acknowledged challenges such as needing to understand current and historical relational dynamics between the patient and carer. This included needing to ensure that the carer was someone the patient was happy to be involved and that they were abiding by data sharing regulations, checking that there were no records of concerns such as neglect due to family care and that the carer was best suited to supporting them.

There might be a family member that came with them to an initial assessment, and they said that they are the first point of contact, but then you find out they actually live abroad. (Professional 15)

### Flexible, Tailored, Cross-System Medication Management Processes

All groups of participants viewed regular medication monitoring incorporating structured medication reviews positively. Primary care was generally seen as having a pivotal role in undertaking this, although there was recognition of the need for coordinated involvement of and communication between professionals from across the healthcare system.

All sides would need to be included. [...] presumably the medication person could be attached to the GP surgery as long as, like communication remained good and you know, everybody knew what was going on. (Carer 1)

Professionals spoke about how deprescribing sometimes highlights conflicting views of medication appropriateness between clinicians. They considered use of multidisciplinary team (MDT) meetings, including the patient and carers, as a valuable means to support decision-making.

In an ideal world, let's say there's [...] you have it almost like a mini-MDT with the pharmacist, the dementia nurse, possibly the GP and the family and the patient. (Professional 3)

However, professionals cited inconsistencies in information technology (IT) systems between organisations and across localities as potentially problematic in achieving a coordinated approach across the system affecting implementation of medication-related decisions/changes including deprescribing.

Maybe some form of technology to enable you to, for us to all share, you know. (Professional 5)

It was evident that a flexible approach to medication management processes is required, given potential for unpredictable changes in health in this group of patients, including cognitive decline. Patients and carers identified a need for accessible support, to facilitate arranging reviews when things change, with a named contact suggested as one way to achieve this.

This is the thing, you don’t need it all the time, [...] you know, it’s just on these one[-off] occasions like that, that you need those doctor’s support, and they ain’t got the time, have they? (Carer 6)

A need to tailor the approach specific to individual patients was also evident, taking into consideration patient and carer resources and combining the medication review with other reviews or appointments.

All participant groups identified a need for proactive, professional-led follow-up during and after deprescribing. Professionals considered it important to undertake deprescribing gradually to ensure it was managed safely, such as slowly tapering medicines and regular checking-in with the patient or carer. Patients and carers highlighted that the follow-up required could be specific to the deprescribed medication.

So sometimes I think a little bit more contact from us in terms of checking in and seeing how [patients] are doing whilst the medications are being reduced. (Professional 15)

[...] and depending on what it was, you might need a blood pressure monitor or a blood test or, you know. (Carer 5)

### Facilitating Deprescribing Discussions

An open, conversation-style discussion explaining the benefits and risks of discontinuing medication was advocated across participant groups. Having adequate time for these discussions was considered essential, with professionals wishing to ensure that they do not overwhelm or rush patients, and patients and carers wanting enough time for full explanations and to ask questions.

Try and be a bit more holistic, but it takes a bit of time to do that. (Professional 8)

You’d want to go into it feeling that you could ask all of the questions that you need to ask and, you know, get the answers that you need and in a format that you need them. (Patient 1)

Trust and continuity of care were perceived as key in facilitating discussions about stopping medicines. Although many patients and carers reported a preference to discuss medications with a doctor, they were open to having these conversations with any trusted and knowledgeable professional. The importance of the professional being aware of their needs and clinical history was highlighted across all participant groups.

Preferably with the one who, who knows you, I think. (Patient 8)

Discussing deprescribing with a knowledgeable professional who was able to answer their questions about medications was important to patients and carers, with professionals agreeing that they should have the combined skills, knowledge, experience and confidence to facilitate deprescribing discussions. Opportunities for training about the importance of deprescribing, specifically focusing on dementia, was advocated by professionals to enable deprescribing practice.

It's about having the right skills and the right knowledge. (Professional 4)

An array of tools and resources were suggested by professionals to support deprescribing discussions. These included risk calculators and visual aids, such as diagrams and pictures, the latter considered useful for patients both during the discussion and to take away. Online resources were perceived as useful for carers outside consultations, although challenging for patients with dementia or MCI.

Online resources I think can be quite difficult to understand for those patients, so that they won't be able to use any of that. (Professional 1)

Professionals also highlighted the value of a safe and comfortable setting to reduce the distress of an unfamiliar environment. Having these discussions in-person was the preference of patients and recognised by professionals as particularly important for patients with dementia or MCI, enabling observation of facial expressions and body language, and depending on the severity of dementia, build rapport with the patient.

I don’t think a telephone conversation [...] it wouldn’t do for me, I don’t think, ‘cause my mind’s not what it was, and I don’t think I could follow that on a telephone conversation as opposed to a face-to-face. (Patient 3)

However, the need to consider the preferred communication preferences and needs of patients and carers was also acknowledged. Indeed, online or telephone communication was considered easier to engage with by some carers.

I don’t mind it [phone contact] because, you know, some of it I don’t mind because you can get it done so much quicker and easier, can’t you? (Carer 8)

### Consideration of Impact of Deprescribing

An individualised, holistic approach to deprescribing was endorsed by many professionals. Key considerations from the perspective of professionals included a person’s capacity and stage of dementia, their priorities in life, their age, overall health and wellbeing, and weighing up the risk and benefit of deprescribing medication.

I don't have hard and fast rules, [...] I would just look at each person as an individual and just think right, is this really adding to this person's quality of life, and it’s sort of that benefit-risk thing. (Professional 2)

Patients and carers as well as professionals regarded medications that impact day-to-day quality of life, medications perceived as key to avoid serious health concerns such as stroke, and medications that control disease, particularly dementia, as important, with perceived importance of a medication influencing willingness to deprescribe.

[...] because I’d then be worried he was going to have another stroke which would make him even more difficult to look after. (Carer 2)

Patients’ views about the importance of individual medicines sometimes differed from professionals. For example, one patient was having a medication prescribed for essential tremor reduced due to professional concerns about its impact on cognition, although from his and his wife’s perspective his tremor had greater impact on day-to-day life, deterioration in cognition viewed as inevitable with dementia:

His hands are the things that, I think, worries him because it is frustrating [...]. So, it is difficult but, equally, his memory is slightly worse but then, I think with that dementia, that is going to happen anyway. (Carer 3)

**Figure 2.**
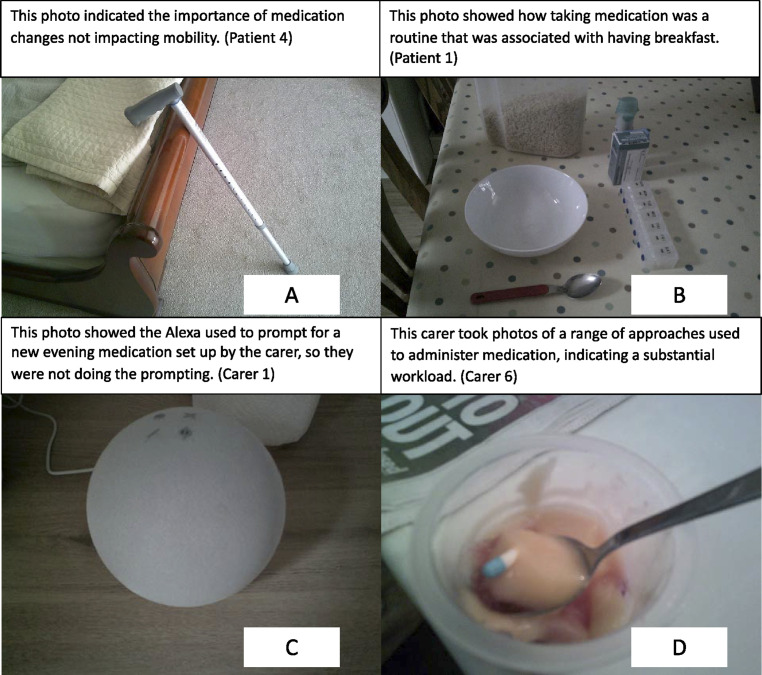
Patient and Carer Photographs

It was also evident that patients might not be able to clearly express their priorities. The quote below shows how one participant struggled to articulate what his photograph of his walking stick (Figure 2(A)) represented. However, his mention of ‘the tablet’ being a reference to medication became apparent across the interview. It was possible to piece together from his transcript the importance of medication changes not negatively impacting his mobility, the photographic data key to understanding this:

The walking stick was the main object of the tablet as far as I was concerned, was walking accurately [...] shows the stick is the dominant treatment. (Patient 4)

Routines underpinned medication management for patients and carers, and were depicted in several participant photographs, in particular the breakfast time routine (Figure 2(B)). Participants identified both positive and negative impacts on the routines of patients and carers when changes were made in medications as a consequence of deprescribing. Although reducing numbers of medications was reported to simplify matters for patients and/or carers, medication changes were also recognised by professionals as potentially confusing for patients. Several considered this particularly challenging for patients without the support of someone to help them remember the changes.

As I said, the routine, if they've got a routine and things are just sort of as they are, they sometimes don't like change. (Professional 7)

However, some carers highlighted that, even when support was available, changes might impact a patient’s independence. Additional carer support could be needed when an embedded routine is altered, although one carer’s photograph highlighted use of technology to provide this support.

It had been forgotten too many times which is why I set-up the Alexa alert. (Carer 1; Figure 2(C))

Additional support due to medication changes also increased carer burden, with carers frequently undertaking substantial medication management work, including negotiating approaches to successfully administer medication (Figure 2(D)). Changes can lead to ordering difficulties, such as order dates getting ‘out of sync’, or a new medication regime not being aligned with the usual routine, for example medication taken at a different time or not being included in pharmacy-prepared pill organisers. Carers valued having support to facilitate process changes with other services such as community pharmacies or home care workers.

[...] and then it all comes out of sync because some, that one’s been increased, and the others aren’t, and I’ve run out. (Carer 3)

## Discussion

This study explored the views and experiences of the three main groups of people involved in deprescribing of medications for people with dementia or MCI, namely people with dementia or MCI themselves, informal carers and healthcare professionals. The findings highlight that deprescribing interventions should involve shared-decision principles and enable the voices of people with dementia or MCI, carers, and professionals from both primary and secondary care to be included. Routine medication management and monitoring processes were considered important to enable deprescribing, with continuity, flexibility, accessibility, and sufficient time for discussions identified as important facilitators of deprescribing interventions for people with dementia or MCI. The findings also provide unique insights into the potential impact of deprescribing, with participant photographs highlighting the effect of deprescribing on routines and processes used in day-to-day medication management at home.

Using the theoretical lens of The Senses Framework, the findings highlight the importance of a relationship-centred approach to deprescribing for people with dementia or MCI, underpinned by care for people with dementia often being provided within triads, consisting of the person with dementia, their informal carer(s) and one or more health and social care professionals (Adams & Gardiner, 2016). The Senses Framework, developed for gerontological practice, proposes that good care can only be delivered when all parties involved experience relationships that promote a sense of *security, belonging, continuity, purpose, achievement* and *significance* (Nolan et al., 2004, 2006). The study findings of a need for face-to-face interactions, ongoing relationships with trusted professionals, and an individualised, structured medication review process align with previous research on deprescribing and shared decision-making in older people with multiple conditions or frailty (Bunn et al., 2018; Radcliffe et al., 2023; Turk et al., 2022). Mapping the findings to The Senses Framework (Table 3) shows that these may help facilitate a sense of *security, continuity* and *belonging* for the person with dementia or MCI but also highlights a wider range of considerations that need addressing.

**Table 3.** Findings Mapped to the Senses Framework

	Person with dementia or MCI	Carer	Professional
Security: To feel safe within relationships	• Safe in the encounter (familiarity – environment, people; pace & style of conversation; trust; written information). • Accessible support. • Knowledgeable professional (competent care, reducing risk from harm).	• Recognising their need for support, such as with processes to help them to provide good care without additional burden.	• Knowledge and system-wide support to inform decision-making. • Work environment supports deprescribing processes required for people with dementia (e.g., enough resources, time, etc.).
Continuity: To feel ‘part’ of things	• Professional who knows the person / context, especially when unaccompanied (recognition and value of personal biography). • Seamless cross-system care.	• Maintaining involvement in care discussions to ensure wishes of people with dementia or MCI are adhered to and inform biography. • Medicines information to assist confidence & competence in care provision.	• Involvement of memory clinic professionals / MDT supports decision-making and potential for exposure to role models. • Cross-system IT to provide good care environment.
Belonging: To experience links and consistency	• Continuity and named contact offer opportunity to form relationships.	• Named contact to confide in and trusted to support the carer with deprescribing / medicines management.	• Feeling supported by others working with people with dementia/MCI through mini-MDT process.
Purpose: To have a personally valuable goal or goals	• Feeling engaged in the process. • Exercise choice by having own priorities and choices recognised.	• Maintaining ‘personhood’ of the individual with dementia or MCI by being involved, seen as a key to informing context of decision-making and being involved in decisions as appropriate.	
Achievement: To make progress towards a desired goal or goals	• Involvement in decision-making and progress towards own goals. • Tailoring to individual needs and avoiding loss of independence sooner than necessary.	• Receiving support with deprescribing / medicines management, such as with processes to help them to provide good care.	• Knowledge and training that is applied in role.
Significance: To feel that ‘you’ matter	• Involvement in decision-making shows they are valued and that they matter; recognising their own goals demonstrates that these have value.	• Recognition of role in medication management, through active involvement in medicines optimisation process.	• Allocation of resource and MDT approaches would indicate that the work is valued and important.

Study participants with dementia or MCI wished to be involved in deprescribing decision-making, reflecting previous research suggesting that people with dementia wish to remain central to decision-making that affects them for as long as possible (Featherstonhaugh et al., 2013). Given the ability to make choices is key to self-determination, this helps provide a sense of both *achievement* and *significance*. However, concerns were expressed by patients and carers about information provided by healthcare professionals and having enough time for discussions and decision-making, something also found in a study investigating the involvement of people with dementia in health and social care decision-making more generally (Tyrrell et al., 2016). Time and appropriate space to discuss options has previously been highlighted as key to support older people to become involved in shared decision-making (Bunn et al., 2018). The study findings suggest that for people with dementia or MCI this is also important so priorities and goals can be clearly established. Given the limited time available in healthcare contexts for the complex decision-making required for deprescribing (Turk et al., 2022), although challenging to achieve, making dedicated time available would provide a sense of *security* for all involved, as would recognition by professionals of the memory recall problems experienced by people with dementia or MCI. Multiple memory aids are widely promoted to support people with dementia (Alzheimer’s Society, 2021), and provision of written information after consultations was considered particularly helpful by patients and carers in this study.

The findings also show the importance of carers, with their involvement offering them a sense of *significance*. Their role providing context to inform discussions was specifically identified, and healthcare professionals are known to require this to support continuity (Turk et al., 2022). Yet, it has been suggested that involving carers in consultations can mean their voice is heard louder than the person with dementia (Tyrrell et al., 2016) and participating professionals expressed the importance of understanding patient-carer dynamics. However, although independence is valued by people with dementia or MCI (Hillman et al., 2023), participants in a study exploring the essence of decision-making in dementia appreciated subtle support that enabled them to continue making decisions independently (Featherstonhaugh et al., 2013). Carers may also validate personhood through support that allows a continued perception of independence (Hillman et al., 2023). Indeed, a tension between independence and interdependence needs to be navigated in dementia care (Lord et al., 2020), the value of interdependence becoming particularly important in the context of cognitive impairment (Keyes et al., 2018). Involvement of the person with dementia or MCI in discussions and decision-making about medications could therefore lead to a sense of *purpose* and *achievement* if this helps them to remain as independent as possible for as long as possible. Given the subtle support that can be provided by carers, it is therefore important that professionals fully explore the impact of medication changes on routines and processes.

The findings suggest the need for flexibility and accessibility, something not specifically identified in a realist review investigating key requirements of deprescribing interventions in primary care for people with frailty (Radcliffe et al., 2023). However, accessible support and having a clear point of contact has previously been proposed as an essential component of dementia care, allowing needs to be managed as they arise (Lord et al., 2020). A 2023 survey of UK informal carers showed that 74% of respondents reported a need for easier ways to manage access to healthcare professionals (Carers UK, 2023). It is important to ensure carers have a sense of *achievement* and *security* in their caring endeavours without additional burden, given that everyday caring experiences are highly correlated with carer wellbeing (Clare et al., 2019). Indeed, the findings showed that carers appreciated support enabling them to navigate the challenges of medication management.

The study findings support previous research highlighting the importance of MDT working (Kaur et al., 2009; Radcliffe et al., 2023) but emphasise the need for improvements in IT-facilitated cross-system communication. A multidisciplinary approach can provide a sense of *belonging* for all involved. Although previous research suggests that relational continuity is the best framework for deprescribing (Radcliffe et al., 2023; Swinglehurst et al., 2023), the study findings suggest that a professional’s knowledge and their ability to answer questions in relation to medication and deprescribing is as important as their knowledge of the patient-carer dyad. Targeted training to increase confidence of primary care professionals in deprescribing has previously been identified as crucial (Radcliffe et al., 2023), something also highlighted by professionals in this study, particularly deprescribing for people with dementia. Having relevant knowledge to apply in practice would help professionals feel a sense of *achievement*. However, education on dementia care more generally may also be beneficial, as a scoping review of dementia care in Europe found limited knowledge among healthcare professionals (Martin et al., 2020). It is important that professionals’ needs, as well as those of people with dementia or MCI and carers, are recognised and addressed to promote good care through a relationship-centred approach (Ryan et al., 2008).

Although the study explored perspectives from people with dementia or MCI, informal carers, and healthcare professionals, these were not gathered as complete triads for the same individual. Instead, the findings reflect the collective perspectives of these groups, sometimes based on different cases. This approach allowed capture of a wide range of experiences and viewpoints, but it does mean that the three-way perspective for a single case was not examined directly. The themes therefore represent commonalities and contrasts across groups rather than integrated narratives from the same patient–carer–professional dyad or triad. While many patient–carer pairs in this study shared broadly similar goals for medication use, concordance is not always present. In some cases, carers and the person with dementia may hold differing or even conflicting priorities. For example, around the use of medications for behavioural or psychological symptoms, where a carer’s preference for reducing challenging behaviours may not align with the patient’s own preferences or sense of wellbeing. Such situations add complexity to shared decision-making and may require sensitive negotiation, clear communication, and facilitation by skilled healthcare professionals to ensure that all voices are heard and that decisions are ethically and clinically appropriate (Poland et al., 2014).

### Strengths and Limitations

The main strength of this study was involving different stakeholders involved in deprescribing discussions, including people with dementia, a group traditionally over-looked in research, their carers, and professionals from a range of disciplines working in primary care and memory clinics. Also, use of photo elicitation allowed the impact of medication changes to be explored in more depth with patients and carers than would otherwise have been possible.

The breadth of insights the study achieved was limited by recruitment of more male than female patients, which does not reflect the population living with dementia, and a lack of ethnic diversity in the sample. The sample included only people with dementia or MCI who had support networks to a greater or lesser extent and may also underrepresent individuals with dementia experiencing more severe neuropsychiatric symptoms, recognising recruiting participants from this group into research is challenging. Although thematic saturation was reached, it is recognised that these findings relate to small sample of participants.

### Implications

Although challenging to achieve, ensuring that all voices in the patient-carer-professional triad are heard requires approaches to care delivery that allow enough time and promote both continuity of care and cross-system working. In practice, it is important that healthcare professionals have the required knowledge to manage deprescribing for people with dementia or MCI, that the impact of deprescribing on the individual and their carers is discussed, and that cognitive impairment is recognised by providing key points from consultations in writing. Box 1 summarises the key practical implications for healthcare professionals. Policy needs to promote provision of accessible support relating to medication management and facilitate working practices such as an MDT approach and cross-system IT. Further research is required to develop and test a primary care deprescribing intervention for community-dwelling people with dementia or MCI using a relationship-centred approach.

Box 1: Key practical implications for healthcare professionals

**Practical implications for healthcare professionals** **–** **applying a** **relationship-** **centred** **approach**

(1) **Maintain continuity with a trusted professional** – Wherever possible, ensure deprescribing discussions are led by someone who already knows the patient–carer dyad and their history. This nurtures trust, security, and a sense of belonging, and makes it easier to align decisions with personal values.
(2) **Provide shared, written records of decisions** – Offer both the patient and carer a clear, accessible written summary of any medication changes, including rationale and agreed follow-up. This respects the voices of all parties, supports memory recall, and reinforces shared understanding.
(3) **Nominate a named relationship contact** – Identify a consistent point of contact for ongoing questions and coordination across settings, so patients and carers feel supported and “known” within the system.
(4) **Include carers as partners in the triad** – Actively invite carers, with patient consent, into deprescribing conversations, recognising their expertise in daily routines, preferences, and health changes. This promotes shared ownership and strengthens the care relationship.
(5) **Tailor pace and style of communication** – Adjust consultations to the patient’s cognitive abilities, preferred style, and emotional needs, using plain language, visual aids, and unhurried dialogue to foster comfort and security in the relationship.
(6) **Respect and protect independence while supporting interdependence** – Before altering regimens, explore how changes will affect daily routines, established habits, and the person’s sense of autonomy. Work with carers to preserve as much independence as possible.
(7) **Recognise and respond to carer needs** – When deprescribing increases carer workload (e.g., changing pill organisers, rescheduling deliveries), offer practical support, guidance, or service coordination to reduce burden and show that their role is valued.
(8) **Coordinate care across the team** – Use multidisciplinary team (MDT) discussions—including memory clinic staff, GPs, pharmacists, nurses, and social prescribers—to make joint decisions, ensuring consistent messages and reinforcing the relationship network around the patient.
(9) **Invest in dementia-specific deprescribing skills** – Seek ongoing training in dementia care and communication, to enhance confidence, empathy, and the ability to navigate the complex relationship dynamics that shape medication decisions.

## Conclusion

This study provides novel insights into deprescribing for people with dementia or MCI, using the lens of people with dementia or MCI, their informal carers and healthcare professionals. Adding to research on deprescribing for older people more broadly, the findings emphasise the importance of using a relationship-centred approach to achieve best deprescribing practice for this group of people. These insights can be used by healthcare professionals to inform their current deprescribing practice, with future work needed to establish primary care deprescribing interventions for people with dementia or MCI. The Senses Framework offers a theoretical and practical framework to achieve this.

## Data Availability

Data generated and/or analysed during this study are included in this published article as supporting quotes and photographs. The full datasets generated and/or analysed during the study (from which these quotes and photographs are taken) are available in the University of Southampton data repository (Andrews et al., 2025). https://doi.org/10.5258/SOTON/D3390.
